# Artificial Intelligence in Burn and Wound Care: Image Analysis, Prediction, and Clinical Integration

**DOI:** 10.1093/jbcr/irag090

**Published:** 2026-06-07

**Authors:** Joshua Khorsandi, Jason Mirharooni, Justin Kahen, Ezzah Tariq, Michael Harouni, Demitri Franzoni, Joshua MacDavid

**Affiliations:** Department of Plastic and Reconstructive Surgery, Kirk Kerkorian School of Medicine at University of Nevada Las Vegas, Las Vegas, NV 89106, United States; Department of Medicine, Florida International University Herbert Wertheim College of Medicine, Miami, FL 33199, United States; Department of Plastic and Reconstructive Surgery, Kirk Kerkorian School of Medicine at University of Nevada Las Vegas, Las Vegas, NV 89106, United States; Department of Plastic and Reconstructive Surgery, Kirk Kerkorian School of Medicine at University of Nevada Las Vegas, Las Vegas, NV 89106, United States; Department of Plastic and Reconstructive Surgery, Kirk Kerkorian School of Medicine at University of Nevada Las Vegas, Las Vegas, NV 89106, United States; Department of Plastic and Reconstructive Surgery, University of Nevada Las Vegas, Las Vegas, NV 89106, United States; Department of Plastic and Reconstructive Surgery, University of Nevada Las Vegas, Las Vegas, NV 89106, United States

**Keywords:** artificial intelligence, wound image analysis, burn-depth assessment, digital health, predictive modeling

## Abstract

Burn injuries and chronic wounds impose a substantial and growing global health and economic burden, particularly in low- and middle-income countries and among aging populations with diabetes, vascular disease, and immobility. Conventional wound assessment depends heavily on visual inspection, manual measurements, and clinician experience, leading to variability in burn-depth estimation, wound sizing, and prognostication. Artificial intelligence, especially deep learning–based computer vision, has emerged as a promising approach to provide objective, reproducible, and scalable evaluation of burns and complex wounds. In this narrative review, we synthesize studies published between 2015 and 2025 focused on 3 domains: image-based wound recognition and segmentation, predictive modeling of outcomes such as healing, graft success, infection, and amputation, and integration of artificial intelligence into telemedicine platforms and smart technologies for remote monitoring. Across multiple datasets, convolutional neural networks achieve segmentation Dice coefficients frequently exceeding 0.85 and burn-depth or tissue-type classification sensitivities above 0.90, while multimodal prediction models reach accuracies and areas under the receiver operating characteristic curve of approximately 0.80-0.95. Early clinical pilots demonstrate the feasibility of embedding artificial intelligence tools into smartphone applications, telehealth workflows, and sensor-enabled dressings. Nonetheless, persistent challenges related to algorithmic bias across skin tones, limited dataset diversity, opaque model behavior, workflow integration, and evolving regulatory frameworks must be addressed before artificial intelligence–enabled wound care systems can be safely and equitably deployed at scale.

## INTRODUCTION

Burn injuries and chronic wounds are major causes of preventable morbidity, mortality, and long-term disability worldwide. Global Burden of Disease analyses estimate that injuries due to fire, heat, and hot substances account for hundreds of thousands of deaths annually, with disproportionate impact in low-resource settings.^1^ Chronic wounds, including diabetic foot ulcers, venous leg ulcers, and pressure injuries, affect ~1%-2% of the population in high-income countries and impose large economic costs, with US expenditures in the tens of billions annually.^2^

Despite advances in surgery, dressings, and adjunctive therapies, assessment remains variable and often subjective. Burn depth, total body surface area (TBSA), and tissue viability are typically estimated visually or with simple bedside tools, and interobserver disagreement can be substantial.^3^ Chronic wound assessment similarly relies on manual planimetry and qualitative tissue descriptions that are prone to error and inconsistent documentation.^4^ As pressures mount to standardize care and extend expertise beyond specialized centers, objective and scalable methods for wound evaluation and outcome prediction are increasingly needed.

Artificial intelligence (AI) has transformed medical imaging and predictive analytics. Deep convolutional neural networks (CNNs) can match or surpass expert performance in tasks such as lesion classification and radiologic interpretation.^5^ In burn and wound care, these approaches are being adapted to automate wound segmentation and classification and to predict outcomes such as healing and infection using multimodal data.^6^ Artificial intelligence may also reduce documentation burden and enable reproducible tracking of wound trajectories.

Telemedicine and mobile health have expanded in burn care, where timely specialist input is often limited by geography, capacity, and disaster-related disruptions. Reviews describe increasing use of store-and-forward images and video consultations for triage and follow-up.^7^ Artificial intelligence–enabled smartphone tools and sensor-based “smart dressings” extend remote assessment and monitoring but raise concerns about safety, equity, and trust.^8^

**Figure 1:**
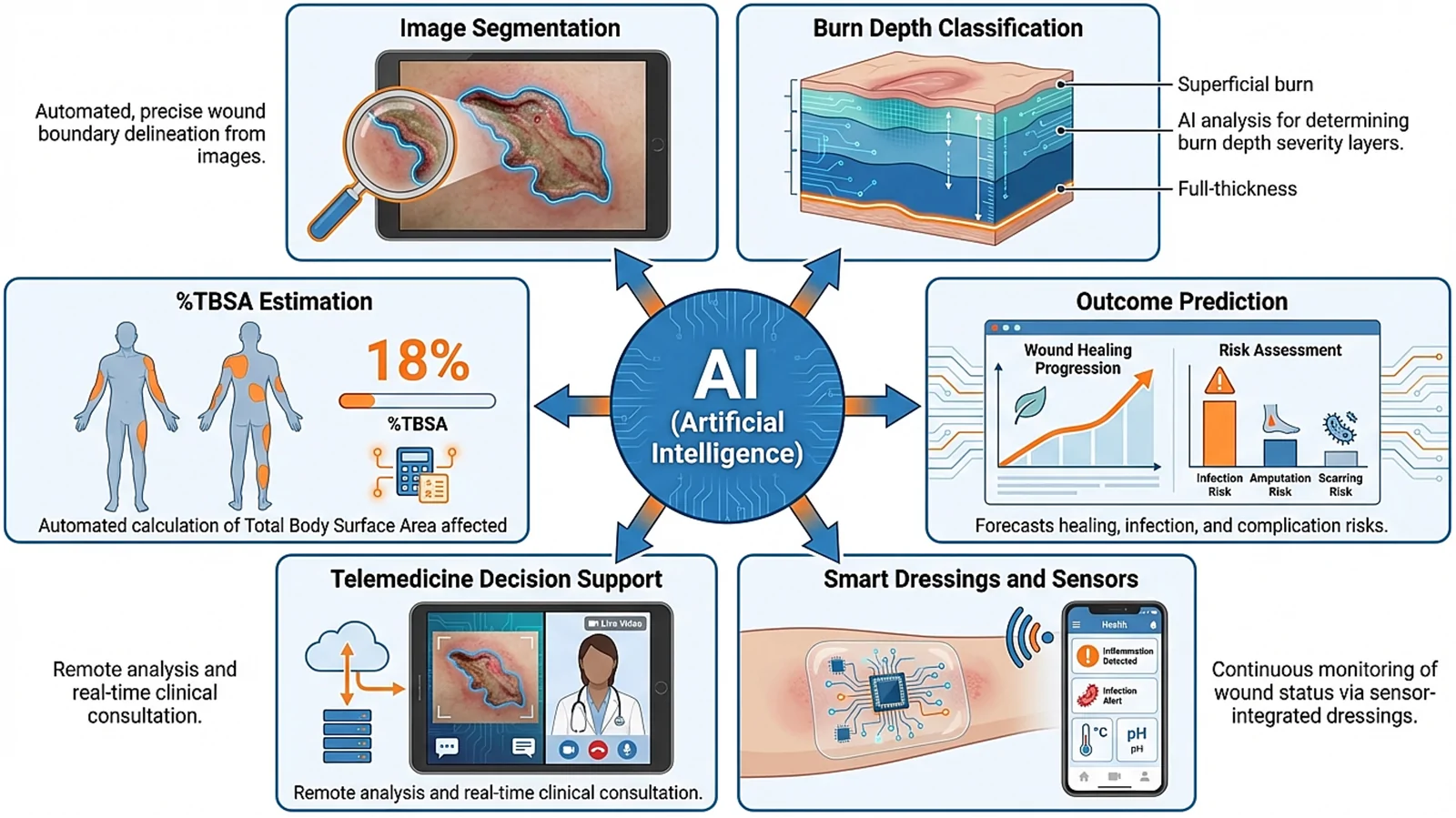
Applications of Artificial Intelligence in Burn and Wound Care, Including Image-Based Wound Segmentation, Burn-Depth Classification, Automated Total Body Surface Area (%TBSA) Estimation, Outcome Prediction, Telemedicine Decision Support, and Integration With Sensor-Enabled Smart Dressings for Remote Monitoring. Image created by authors using Figure Labs. Abbreviations: AI, artificial intelligence; TBSA, total body surface area.

Artificial intelligence’s promise is 2-fold: improving the accuracy and efficiency of wound assessment and expanding decision support beyond tertiary centers (Figure 1). Barriers include bias from nonrepresentative datasets (including limited darker-skin representation), limited generalizability across devices and settings, opaque model behavior, workflow integration challenges, and evolving regulatory expectations.^9^

Accordingly, this narrative review synthesizes literature from 2015 to 2025 across 3 domains: (1) image-based wound recognition and segmentation, (2) predictive modeling of clinically meaningful outcomes, and (3) integration of AI tools into telemedicine platforms and smart technologies. We highlight cross-cutting issues in dataset diversity, explainability, clinical workflow integration, and ethical/regulatory considerations required for safe and equitable deployment of AI-enabled wound care systems. This review is intended for clinicians, translational researchers, and multidisciplinary stakeholders in burn and wound care who seek a clinically oriented overview of the current capabilities, limitations, and future translational potential of AI, rather than a purely technical analysis of computational model development.

## METHODS

This structured narrative review synthesized literature on AI in burn and cutaneous wound care. A structured narrative review methodology was selected rather than a formal systematic review because the literature on AI in burn and wound care remains highly heterogeneous with respect to study design, datasets, imaging modalities, AI architectures, outcome measures, and stages of clinical translation. The objective of this review was therefore to provide a broad synthesis of emerging applications, translational challenges, and future directions across multiple interconnected domains rather than to answer a single narrowly defined question suitable for meta-analysis.

PubMed, Scopus, and Web of Science were searched for English-language articles published from January 1, 2015 through June 30, 2025. Database-specific search strings combined burn/wound terms (“burn,” “thermal injury,” “chronic wound,” “diabetic foot ulcer,” “pressure ulcer,” and “venous leg ulcer”) with AI terms (“artificial intelligence,” “machine learning,” “deep learning,” “convolutional neural network,” “computer vision,” “segmentation,” “classification,” “prediction,” “telemedicine,” and “smart dressing”). Boolean operators and truncation were used, and search strings were adapted per database. Reference lists of relevant studies were screened for additional articles.

Titles/abstracts were screened, followed by full-text assessment. We included original research or systematic reviews applying an AI/machine learning (ML) method to imaging (recognition, segmentation, and depth/tissue classification), outcome prediction, or decision support in burns or clinically relevant cutaneous wounds (human or clinically relevant preclinical datasets). We excluded editorials/commentaries without original data, conference abstracts without full manuscripts, studies focused solely on non-cutaneous wounds, and reports using only traditional statistical models without an AI/ML component. For overlapping datasets, the most comprehensive or most recent report was prioritized. Using these methods, a total of 35 sources were included in the final narrative synthesis.

Included studies were organized into 3 domains: (1) image-based wound recognition and segmentation; (2) predictive modeling of outcomes (healing trajectory, complications, graft success, infection, and amputation risk); and (3) integration into telemedicine, mobile health, or smart technologies (eg, sensor-enabled dressings and connected devices). For each study, we extracted clinical context, dataset characteristics (sample size, modality, centers, geography, and reported skin tone/etiology distribution when available), reference standard (eg, histopathology, laser Doppler imaging, expert consensus, or longitudinal outcomes), model approach, and key performance metrics. We also noted labeling procedures and whether interrater reliability was reported.

We recorded performance measures as reported (eg, Dice/intersection-over-union for segmentation; accuracy, sensitivity/specificity, and AUROC for classification/prediction) and documented validation strategy (cross-validation, held-out testing, or external validation). Where available, comparisons with clinician performance were recorded. Translational features were qualitatively coded (workflow integration, user interface descriptions, and clinician/patient acceptability) along with reporting of dataset diversity, interpretability methods, and ethical/regulatory considerations (eg, bias, consent, software-as-a-medical-device guidance). Given heterogeneity across designs and outcomes, meta-analysis was not performed; findings were synthesized narratively across domains.

## RESULTS

### Image-based wound recognition and segmentation

The search identified a growing body of work applying deep learning–based computer vision to the automated segmentation and characterization of burn and chronic wound images. Early efforts adapted generic CNN architectures such as U-Net and Mask R-CNN to delineate wound boundaries and distinguish wound from background skin, achieving promising results in diabetic foot ulcer and mixed chronic wound datasets.^10^ More recent studies have introduced specialized architectures, including dual-attention U-Nets, feature pyramid networks, and multi-scale ensembles, which report Dice similarity coefficients in the range of 0.85-0.93 for wound segmentation across heterogeneous imaging conditions.^11^ Meta-analytic syntheses of deep learning–based chronic wound segmentation confirm that, when evaluated on adequately sized test sets, state-of-the-art models frequently achieve Dice coefficients above 0.85, with best-performing systems approaching or exceeding 0.90.^12^

In burn care, several groups have developed CNN-based methods for automatic segmentation of burn regions and estimation of TBSA from 2-dimensional or 3-dimensional images. Investigators in a study published in the *Journal of Medical Internet Research* proposed a deep learning framework that segments burn wounds and calculates TBSA on a pixel-wise basis, reporting performance comparable to that of experienced clinicians in synthetic and clinical scenarios.^13^ A multicenter study using on-site smartphone-captured color images reported average Dice coefficients of approximately 0.85 for segmentation of burn-depth regions and body parts, with an *R*^2^ of 0.91 for TBSA estimation compared with expert-derived measurements.^14^ A comprehensive review of AI in wound repair noted that, among burn wound segmentation models, a Mask R-CNN architecture with ResNet-101 backbone achieved a Dice coefficient near 0.95 in a single-center dataset, outperforming U-Net–based approaches and underscoring the potential for highly accurate automated delineation of burn areas when sufficient training data are available.^6^ Pediatric-specific work, such as the Pediatric BurnNet framework, has shown that attention-augmented deep learning can simultaneously perform segmentation and severity classification in images acquired under varied real-world conditions, although training remains limited by smaller pediatric datasets.^15^

Several studies extend segmentation beyond simple wound boundaries to tissue-type classification, aiming to quantify proportions of granulation tissue, slough, eschar, and epithelialization. Deep learning models trained on annotated multi-tissue datasets achieve weighted-average Dice scores in the low 0.80s, with misclassification most frequent at tissue interfaces and in images with marked lighting variation or occluding dressings.^16^ These techniques offer the prospect of objective, longitudinal tracking of wound bed composition that could be correlated with healing trajectories and used to guide debridement decisions.

### Burn-depth and wound-type classification

Complementing segmentation efforts, numerous studies have trained CNNs to classify burn depth and to stage or etiologically classify chronic wounds. In burns, investigators have developed models that distinguish superficial, partial-thickness, and full-thickness burns using color photographs, multispectral images, or thermal and hyperspectral modalities.^17^ Reported sensitivities and specificities for identifying surgically significant depth (ie, burns likely to require grafting) commonly exceed 0.85, with some deep learning models surpassing 0.90 in internal validation.^14^ However, performance often deteriorates in external datasets acquired with different cameras, lighting conditions, or patient populations, highlighting risks of domain shift.

In addition to conventional color-image analysis, emerging work has explored multispectral and hyperspectral imaging combined with ML for burn-depth assessment and healing prediction.^18^ By capturing optical information beyond the visible spectrum, these technologies may better characterize tissue perfusion, oxygenation, and viability than standard photography alone. Recent investigational systems applying CNNs and related AI approaches to multispectral imaging data have shown promise in differentiating burns likely to heal conservatively from those requiring operative intervention, though prospective validation and broader clinical integration remain limited.^18^

Artificial intelligence models have also been trained to classify chronic wound etiology (eg, diabetic foot ulcer, venous leg ulcer, or pressure injury) and to stage pressure injuries based on smartphone photographs.^10^ These systems report classification accuracies in the 0.85-0.95 range and can support nonexpert caregivers by flagging images that merit specialist review.^8^ Combined segmentation and classification pipelines have demonstrated that architectures optimized for one task (eg, boundary delineation) can be extended to tissue or severity classification with additional training, though performance may be constrained by the limited size and diversity of labeled wound datasets.

Across both burn and chronic wound applications, a recurring limitation is the underrepresentation of darker skin tones and certain wound etiologies (eg, electrical burns, chemical burns, or atypical vasculitic ulcers). General and dermatology-focused analyses reveal that many medical imaging datasets used for AI development are drawn disproportionately from lighter-skinned populations and from a narrow set of geographic regions, raising concerns that published performance metrics may not generalize to patients with more heavily pigmented skin or from under-resourced settings.^9^

### Predictive modeling of wound outcomes

Beyond static image interpretation, AI has been applied to prognostic modeling in both burn and chronic wound care. In burn populations, ML models incorporating demographic variables, comorbidities, TBSA, inhalation injury, and early laboratory values have been developed to predict mortality, sepsis, and other complications. Studies using artificial neural networks and ensemble methods report accuracies of approximately 90%-96% in predicting mortality in tertiary-center cohorts, outperforming traditional severity scores such as Baux and the Abbreviated Burn Severity Index.^19^ A streamlined multicenter model based on only 6 admission variables achieved strong discriminative performance for early sepsis risk in more than 6000 patients with burn injuries, highlighting the feasibility of parsimonious, interpretable models tailored for real-world deployment.^20^ More recently, interpretable web-based prediction tools for sepsis and mortality in patients with extensive burn injuries have been developed using gradient-boosted decision trees and Shapley value–based explanations, enabling clinicians to visualize how individual features contribute to risk estimates.^21^

For chronic wounds, ML models have been used to predict healing times, recurrence, infection, and amputation risk, often by integrating clinical variables with image-derived features. Systematic reviews of AI methods for diagnostic and decision-making assistance in chronic wounds report that logistic regression, random forests, support vector machines, and deep learning architectures achieve areas under the curve commonly in the 0.80-0.90 range for predicting healing within defined time windows, with some models approaching 0.95 in internally validated cohorts.^4^ In diabetic foot ulcer populations, multiple studies have developed prediction models for minor and major amputation, using both traditional clinical risk factors and imaging or perfusion data. Some gradient-boosting and explainable ML models report accuracies above 0.90 and provide ranked feature importance, consistently identifying infection severity, inflammatory markers, neuropathy, vascular status, and ulcer duration as key predictors.^22^ Meta-analytic work suggests that, across studies, ML offers at least moderate diagnostic accuracy for predicting diabetes-related amputation, though heterogeneity in model quality and risk of bias remains substantial.^19^

Skin graft–related outcomes have also attracted interest. While much of the literature on split-thickness skin graft loss focuses on conventional risk factor analysis, exploratory work in animal models has demonstrated that ML applied to laser speckle imaging can predict graft failure with clinically useful accuracy before gross necrosis is apparent, potentially enabling earlier intervention.^23^ More recently, AI-based strategies have been applied to modeling graft expansion patterns and optimizing meshing designs, although these techniques are still in early development stages.^24^  Table 1 provides an overview of representative AI applications across burn and chronic wound care, summarizing datasets, modeling approaches, and key performance findings spanning image segmentation, TBSA estimation, classification, and outcome prediction.

**Table 1:** Summary of AI Applications and Key Findings Across Burn and Wound Care Domains

Domain	Data/setting	AI approach	Key finding(s)
Burn image segmentation + depth labeling	Labeled burn photos with depth regions plus hand/palm datasets.	U-Net vs Mask R-CNN with ResNet backbones.	Mask R-CNN (ResNet101) achieved a Dice coefficient of 0.9496 for burn wound segmentation and outperformed U-Net for burn segmentation and classification.^13^
Burn %TBSA estimation support	Standardized photos of multiple body regions from one patient and surgeon estimates.	Mask R-CNN segmentation + pixel-based %TBSA calculation.	Mask R-CNN had a smaller average deviation from ground truth (0.115% TBSA) than burn surgeons (0.45%-1.14% TBSA) in the study test scenario.^13^
Acute burn detection + “needs surgery” classification	1105 burn images plus 536 background images, with analysis by Fitzpatrick skin type groups.	Commercial CNN workflow trained for (1) wound identification and (2) surgery-needed classification.	The wound identifier detected an average of 87.2% of wound areas in the test set, and the wound classifier achieved an AUC of 0.885, with performance differences across Fitzpatrick skin type groupings.^17^
Burn surgical candidacy prediction (multimodal)	Burn-center cohort data with both images and structured clinical variables.	Multimodal deep learning combining image and clinical features.	The study demonstrated feasibility of predicting burn surgical candidacy using multimodal deep learning intended for triage and decision support rather than image-only assessment.^25^
Chronic wound healing time prediction	Chronic wound populations with longitudinal outcomes.	Supervised ML models predicting time-to-heal.	The study supported the use of ML to predict chronic wound-healing time to enable earlier risk stratification and care planning.^26^
Diabetic foot ulcer amputation risk (multicenter, explainable ML)	Multicenter DFU cohort with external validation.	ML model with interpretability methods.	The external validation AUC was 0.843 (95% CI 0.758-0.928), supporting clinically relevant discrimination for amputation risk across sites.^22^

Abbreviations: AI, artificial intelligence; AUC, area under the receiver operating characteristic curve; CNN, convolutional neural network; DFU, diabetic foot ulcer; ML, machine learning; RCNN, region-based convolutional neural network; TBSA, total body surface area; U-Net, convolutional neural network architecture for image segmentation.

### Telemedicine, mobile health, and smart technologies

A third major domain involves the integration of AI into telemedicine workflows, mobile applications, and sensor-enabled dressings. Telehealth has a long history in burn care, but recent reviews document an acceleration in teleconsultations, remote triage, and video-based follow-up, facilitated by smartphones and secure platforms.^7^ Studies from high- and middle-income countries report that teleburn consultations can reduce unnecessary transfers, improve accuracy of triage decisions, and shorten time to specialist input, although technical and organizational barriers persist.^7^

Artificial intelligence is increasingly embedded within these remote workflows. Prototype smartphone applications for burn assessment combine standardized data entry based on Advanced Burn Life Support principles with computer vision modules that estimate burn size and depth from photographs, providing automated suggestions for fluid resuscitation and referral level.^27^ Other mobile tools use AI to assist nonexpert caregivers in staging pressure injuries or recognizing high-risk chronic wounds in nursing home and community settings.^8^ These applications often emphasize intuitive user interfaces and real-time feedback, but most remain in pilot or early clinical testing, with limited evidence of long-term adoption or impact.

Smart dressings and wearable devices represent an emerging frontier. Sensor-enabled bandages incorporating impedance, temperature, pH, or oxygenation sensors can generate continuous data streams indicative of local perfusion, inflammation, or bacterial load. When coupled with AI algorithms, these platforms have been shown in preclinical and early clinical studies to detect changes predictive of wound deterioration or infection and, in some cases, to modulate therapeutic interventions such as electrical stimulation or controlled drug release.^28^ Remote healthcare frameworks highlight that AI-driven analytics can help prioritize alerts, flag high-risk patients, and support resource allocation in home care and rural settings, though regulatory approvals for fully autonomous systems remain limited.^29^

Across telemedicine and smart-technology studies, investigators consistently underscore unresolved challenges, including workflow integration, interoperability with electronic health records, cybersecurity, data governance, and the need for robust evidence from prospective trials or implementation studies.^7^

### Cross-cutting limitations

Despite encouraging metrics, key limitations persist. Many datasets are small, single-center collections captured with specific devices and protocols, increasing risks of overfitting and weak generalizability.^10^ External validation across institutions, cameras, and populations remains uncommon, and performance often drops with shifts in lighting, framing, wound appearance, and practice patterns.^12^

Ground truth is also inconsistent. For burn depth, histopathology and laser Doppler imaging are stronger standards, yet many studies rely on single-expert labels or consensus without independent confirmation, introducing label noise that can cap performance.^13^ Chronic wound datasets similarly depend on clinician-drawn outlines and subjective tissue-type labels, with limited reporting of interrater reliability.^10^ Finally, fairness assessment is often missing: broader work shows underrepresentation of darker skin tones and marginalized groups in training data, but few wound studies report Fitzpatrick types, race/ethnicity, or socioeconomic factors, and only a minority perform subgroup or fairness-metric analyses, raising risk of amplifying inequities without safeguards.^9^

## DISCUSSION

This structured narrative review demonstrates that AI applications in burn and wound care have evolved rapidly over the past decade, mirroring broader trends in medical imaging and predictive analytics. Deep learning–based segmentation and classification models now achieve performance metrics that rival or exceed those of human experts in controlled settings, and prognostic models increasingly integrate complex clinical and imaging data to estimate risk of adverse outcomes. Yet the field remains characterized by small, siloed datasets, limited external validation, and insufficient attention to fairness, explainability, and clinical integration.

### Sources of bias, error, and limited generalizability

Mechanistically, several factors contribute to the fragility and potential bias of wound-related AI systems. First, image acquisition is inherently variable. Wound photographs are captured using different cameras and smartphones, with heterogeneous resolutions, color balance, focal distances, and lighting conditions. Backgrounds range from clean drapes to cluttered clinical environments or home settings, while the presence of dressings, exudate, and motion artifacts further complicates analysis.^10^ Convolutional neural networks are highly sensitive to these low-level visual changes, and unless models are trained on diverse, augmented datasets or explicitly domain-adapted, performance may degrade markedly when deployed outside the development environment.

Second, annotation and ground truth imperfections introduce both random and systematic error. In burn-depth classification, expert disagreement is common, and even sophisticated methods—such as laser Doppler imaging or indocyanine green angiography—provide only probabilistic assessments of tissue viability.^3^ When labels are noisy or inconsistent, deep learning models may learn to reproduce idiosyncratic patterns in the training set rather than capturing robust pathophysiologic features, reducing generalizability. Similar concerns apply to chronic wound segmentation and tissue typing, where boundaries between tissue classes are often ambiguous and subject to interobserver variability.^10^

Third, dataset composition frequently reflects structural inequities. Analyses of dermatology and medical imaging AI have shown that training datasets disproportionately include lighter-skinned individuals and patients from a small number of well-resourced institutions, resulting in models that perform worse on darker skin tones and underrepresented populations.^9^ There is little reason to believe that existing burn and wound datasets are exempt from these biases; in fact, anecdotal reports and single-center experiences suggest substantial gaps in representation. Underrepresentation of severe burns, pediatric cases, electrical and chemical injuries, and atypical chronic wounds further constrains model applicability across the full spectrum of clinical presentations.

Finally, outcome ground truth in prognostic modeling is often incomplete or confounded. Healing, graft success, infection, and amputation are influenced by numerous factors, including comorbidities, vascular status, infection control, adherence, and social determinants such as housing stability and access to care.^4^ Retrospective datasets may not capture these variables adequately, leading models to learn proxies that may be unstable or reflect institutional practice rather than underlying biology. Moreover, missing data, informative censoring, and competing risks can bias estimates of healing times or complication rates if not carefully accounted for in model development and evaluation.

### Ethical and governance considerations

Ethical evaluation of wound AI can follow Beauchamp and Childress: autonomy, beneficence, nonmaleficence, and justice.^30^ Systems should improve accuracy/efficiency without misclassification or automation bias, be transparent to clinicians/patients, and perform equitably across skin tones and settings.^9^ Regulators now emphasize Good Machine Learning Practice (FDA, Health Canada, MHRA) and lifecycle oversight, while the EU AI Act/medical-device rules add conformity assessment and post-market monitoring.^31^ Reporting standards (TRIPOD + AI, CONSORT-AI, and SPIRIT-AI) should also guide study design.^32^

### Promise of AI for triage, prediction, and resource optimization

Despite these caveats, the transformative potential of AI in burn and wound care is substantial. Automated image analysis can standardize wound measurements, reduce documentation burden, and provide objective baselines for tracking healing over time.^10^ In burn triage, AI-enabled smartphone tools could help first responders and nonspecialist clinicians more accurately estimate TBSA, identify burns requiring transfer, and initiate appropriate early management, thereby optimizing use of limited burn center resources and potentially improving outcomes in mass casualty events.^27^

Predictive models can support individualized care planning by estimating the likelihood and timing of wound healing, graft success, infection, and amputation, enabling earlier interventions for high-risk patients and more efficient use of advanced therapies and multidisciplinary services.^4^ At the system level, AI-driven analytics applied to large wound registries and administrative data could inform resource allocation, quality improvement initiatives, and policy decisions.

Telemedicine and smart technologies extend these benefits beyond the hospital, supporting remote monitoring and timely intervention for patients in rural or underserved settings, reducing travel burdens, and potentially preventing avoidable hospitalizations.^7^ By enabling earlier detection of deterioration or infection and facilitating proactive outreach, AI-enhanced remote care may reduce disparities in access to specialist services and improve equity in wound outcomes, provided that systems are designed with inclusivity and affordability in mind.

Importantly, although many AI systems demonstrate promising diagnostic and predictive performance, the overall level of clinical evidence remains limited. Most published studies are retrospective, single-center investigations or technical feasibility studies with limited external validation and few prospective implementation trials. Consequently, many AI applications in burn and wound care should currently be viewed as investigational or adjunctive decision-support tools rather than established standards of care. Wider clinical adoption will require multicenter prospective validation, assessment across diverse patient populations and skin tones, demonstration of clinical utility and cost-effectiveness, and continued regulatory oversight before routine incorporation into practice can be recommended.

### Strategies to advance safe and equitable translation

To move from promising prototypes to safe, trusted, and widely adopted AI-enabled wound care systems, several actionable strategies are needed.

First, there is an urgent need for representative, high-quality datasets that capture the diversity of patients and wound presentations encountered in real-world practice. Collaborative, multi-institutional efforts—potentially linked with initiatives such as the WHO Global Burn Registry—could support the development of large, standardized image and clinical datasets with detailed annotation, including skin tone, wound etiology, and social determinants of health.^31^^–^^33^ Appropriate governance, consent, and privacy protections are essential, especially when leveraging cloud-based storage and cross-border data sharing.

Second, rigorous external validation across multiple centers, devices, and populations should become standard practice before clinical deployment. This includes not only traditional accuracy metrics but also systematic evaluation of performance by skin tone, demographic subgroup, and care setting. Reports of such evaluations should adhere to updated reporting standards such as TRIPOD + AI and, for interventional trials of AI systems, CONSORT-AI and SPIRIT-AI.^33^

Third, bias evaluation and mitigation tools must be integrated into the AI lifecycle. This may involve proactive sampling strategies to balance datasets, algorithmic fairness constraints during training, and continuous monitoring for emergent disparities in model performance after deployment.^9^ Transparent reporting of dataset composition, labeling processes, and fairness analyses will enable independent scrutiny and foster trust.

Fourth, human-in-the-loop and clinician-in-the-loop approaches offer a pragmatic path to harness AI while maintaining expert oversight. In such systems, AI models provide measurements, risk scores, or prioritized worklists, but final decisions rest with clinicians who can override or question algorithmic outputs.^34^ These approaches align with regulatory expectations for human accountability and may facilitate gradual adoption as clinicians build familiarity and calibrate their trust in AI tools.

Finally, robust post-deployment monitoring frameworks are needed to detect performance drift, unintended consequences, and fairness issues in real-world use.^35^ Health systems deploying AI-enabled wound care tools should establish clear governance structures, including key performance indicators, thresholds for action, and mechanisms for clinician and patient feedback. Iterative model improvement and, when appropriate, decommissioning should be viewed as integral to the total product lifecycle rather than as optional add-ons. Table 2 summarizes the major application domains of AI in burn and wound care, outlining the associated clinical tasks, typical data inputs, commonly used modeling approaches, and the current maturity of the supporting evidence.

**Table 2:** Major Application Areas of AI in Burn and Wound Care

Application domain	Clinical task	Typical data inputs	AI methods used	Current evidence maturity	Representative reference
Burn and wound segmentation	Automated wound boundary and area delineation	2D color images, 3D reconstructions	CNNs (U-Net, Mask R-CNN, attention U-Net)	Moderate–high (multiple validation studies)	Chang et al.^13^
Burn-depth classification	Identification of superficial vs full-thickness burns	Color, thermal, multispectral images	CNN classifiers, ensemble models	Moderate (mostly internal validation)	Boissin et al.^17^
TBSA estimation	Automated %TBSA calculation	Annotated body images	Segmentation + pixel-based estimation	Moderate (limited real-world testing)	Chang et al.^13^
Chronic wound tissue typing	Granulation, slough, eschar classification	Annotated wound photographs	Multi-class CNNs	Low–moderate (dataset-dependent)	Berezo et al.^26^
Outcome prediction (burns)	Mortality, sepsis, complications	Demographics, TBSA, labs, images	ANN, gradient boosting, multimodal DL	Moderate (few external validations)	Rambhatla et al.^25^
Outcome prediction (chronic wounds)	Healing time, amputation risk	Clinical + imaging data	RF, SVM, explainable ML	Moderate (heterogeneous quality)	Tao et al.^22^
Telemedicine decision support	Remote triage and follow-up	Smartphone images, questionnaires	Embedded CNN pipelines	Low–moderate (pilot studies)	Boissin et al.^17^

Abbreviations: AI, artificial intelligence; ANN, artificial neural network; CNN, convolutional neural network; DL, deep learning; ML, machine learning; RF, random forest; SVM, support vector machine; TBSA, total body surface area; U-Net, convolutional neural network architecture for image segmentation.

## CONCLUSION

Artificial intelligence is becoming an important tool in burn and wound care, enabling objective image analysis, outcome prediction, and scalable support for remote monitoring and triage. Deep learning models now approach expert performance in tasks such as segmentation and classification, while prognostic systems estimate individualized risks for healing, graft success, infection, and amputation. Integrated into telemedicine platforms and smart dressings, these tools can extend specialist expertise to underserved settings, standardize care, and optimize resource use. Yet meaningful clinical adoption remains challenging due to algorithmic bias from nonrepresentative datasets, limited external validation, opaque model behavior, and evolving regulatory expectations. Addressing these barriers through representative data, rigorous validation, bias mitigation, clinician-in-the-loop design, and ongoing monitoring can ensure that AI complements—rather than replaces—expert judgment and advances timely, precise, and equitable wound care.

## Notes

Author contributions: Joshua Khorsandi (Project administration, Writing—original draft [lead]), Jason Mirharooni (Writing—review & editing [equal]), Justin Kahen (Investigation, Project administration [supporting]), Ezzah Tariq (Methodology [equal]), Michael Harouni (Formal analysis [equal]), Demitri Franzoni (Supervision [equal]), and Joshua MacDavid (Writing—review & editing [supporting])

Funding: This research received no external funding.
